# Enhancement of fatty acid degradation pathway promoted glucoamylase synthesis in *Aspergillus niger*

**DOI:** 10.1186/s12934-022-01966-3

**Published:** 2022-11-15

**Authors:** Jie Qi, Xianzun Xiao, Liming Ouyang, Chenghan Yang, Yingping Zhuang, Lixin Zhang

**Affiliations:** State Key Laboratory of Bioreactor Engineering, East China University of Science and Technology, Shanghai, 200237 People’s Republic of China

**Keywords:** Metabolic engineering, Microbial cell factories, *Aspergillus niger*, Glucoamylase, Fatty acids degradation, Chemostat, Tet-on.

## Abstract

**Background:**

Our recent multi-omics analyses of glucoamylase biosynthesis in *Aspergillus niger* (*A. niger*) suggested that lipid catabolism was significantly up-regulated during high-yield period under oxygen limitation. Since the catabolism of fatty acids can provide energy compounds such as ATP and important precursors such as acetyl-CoA, we speculated that enhancement of this pathway might be beneficial to glucoamylase overproduction.

**Results:**

Based on previous transcriptome data, we selected and individually overexpressed five candidate genes involved in fatty acid degradation under the control of the Tet-on gene switch in *A. niger*. Overexpression of the *fadE*, *fadA* and *cyp* genes increased the final specific enzyme activity and total secreted protein on shake flask by 21.3 ~ 31.3% and 16.0 ~ 24.2%, respectively. And a better inducible effect by doxycycline was obtained from early logarithmic growth phase (18 h) than stationary phase (42 h). Similar with flask-level results, the glucoamylase content and total extracellular protein in engineered strains OE-*fadE* (overexpressing *fadE*) and OE-*fadA* (overexpressing *fadA*) on maltose-limited chemostat cultivation were improved by 31.2 ~ 34.1% and 35.1 ~ 38.8% compared to parental strain B36. Meanwhile, intracellular free fatty acids were correspondingly decreased by 41.6 ~ 44.6%. The metabolomic analysis demonstrated intracellular amino acids pools increased 24.86% and 18.49% in two engineered strains OE-*fadE* and OE-*fadA* compared to B36. Flux simulation revealed that increased ATP, acetyl-CoA and NADH was supplied into TCA cycle to improve amino acids synthesis for glucoamylase overproduction.

**Conclusion:**

This study suggested for the first time that glucoamylase production was significantly improved in *A. niger* by overexpression of genes *fadE* and *fadA* involved in fatty acids degradation pathway. Harnessing the intracellular fatty acids could be a strategy to improve enzyme production in *Aspergillus niger* cell factory.

**Supplementary Information:**

The online version contains supplementary material available at 10.1186/s12934-022-01966-3.

## Introduction

*Aspergillus niger* (*A. niger*) is an important microbial cell factory. Due to its excellent protein expression and secretion ability, strong environmental adaptability and mature fermentation process, it is widely used in the production of organic acids and enzyme preparations such as glucoamylase [1–3]. Glucoamylase is a commercial biocatalyst with extremely high demand in the food and beverage industry. This enzyme was first discovered in *A. niger* in 1970 s, and its highest industrial yield has reached 30 g/L till the moment [4]. At present, the construction of high-copy glucoamylase gene (*glaA*) overexpression strains [5] and optimization of fermentation medium [6], process and conditions [7–9] such as optimization of mycelial morphology, temperature, pH, aeration and stirring are all common strategies to improve the industrial glucoamylase production. In addition, oxygen limitation is also considered as a highly effective strategy because of the high yield of glucoamylase to substrate and few by-products at late stage of fermentation under this condition [10]. In brief, most of these studies focused on improvement of glucoamylase production via genetic engineering and fermentation process optimization, but rarely via metabolic engineering.

In recent years, the integration of process multi-omics data analysis largely extends the systematic understanding of the efficient production mechanism of *A. niger* and indicates directions for rational global optimization of strain performance by genetic modification and process regulation. For example, Cao et al. [11] predicted limited amino acids for glucoamylase synthesis through multi-omics analysis, and increased glucoamylase production in *A. niger* by improving their biosynthesis under oxygen limitation. In another study, based on multi-omics analysis, researchers speculated low NADPH availability as a limiting factor for higher glucoamylase production, and improved enzyme yield by overexpressing different NADPH supply pathways [12].

Interestingly, multi-omics integrative analysis of glucoamylase fermentation process in *A. niger* in our lab [13] showed that fatty acids degradation and precursor amino acids synthesis were elevated under oxygen limitation, whereas fatty acid synthesis was diminished. While it is well known that lipids, as essential components of cell membranes, play an important role in many processes such as cell signaling, energy supply and cell growth [14]. Most previous studies of lipid metabolism in fungi focus on the enhancement of production of lipids, but rarely on its metabolic impact to other cell factory products. Wang et al. reported the degradation of triacylglycerols (TAGs), which accumulates in primary metabolism, could channel carbon flux from both intracellular TAGs and extracellular substrates into polyketide biosynthesis during stationary phase in *Streptomyces* [15]. It indicates the regulation of lipid metabolism could relocate cell resource to the synthesis of secondary metabolism products. Thus, we speculated that the enhancement of fatty acids degradation pathway might further improve the enzyme yield.

Herein, we selected candidate genes involved in fatty acids degradation based on previous transcriptome analysis of glucoamylase production in *A. niger* under oxygen limitation and constructed overexpression strains of these genes under the control of Tet-on system. Then all overexpression strains were cultivated in shake flask to screen out the strains with the ability of promoting glucoamylase synthesis. At last, the screened strains were preliminarily explored this strategy’s global impact on intracellular metabolism via maltose-limited chemostat culture in bioreactor.

## Materials and methods

### Strains

B36 is a high-yielding glucoamylase engineered strain with multi-copy glucoamylase coding gene *glaA* and derived from N402 (ATCC64974) [5]. YS20.2, a uridine auxotrophic derivative (*pyrG*^−^) of B36, was used as the parental strain for construction of all overexpression mutants in this study [12]. All strains used in this study are listed in Table 1.

**Table 1:** Strains and plasmids used in this study

Strains or plasmids	Background strain, relevant genotype or characteristics	Function	Source
Strains			
*Escherichia coli* DH5α	F^−^, Φ80*lac*ZΔM15, Δ(*lac*ZYA-*arg*F) U169, *rec*A1, *end*A1, *hsd*R17(r_k_^−^, m_k_^+^), *pho*A, *sup*E44, *thi*-1, *gyr*A96, *rel*A1, λ^−^	For plasmid construction and ampification	Sanggon, Shanghai, China
*Aspergillus niger* B36	High glucoamylase-producing strains with multiple copies of *glaA*	Reference strain	[5]
YS20.2	B36, *pyrG*^*−*^	Parental strain for *A. niger* transformation	[12]
OE-*fadE*	YS20.2, Tet-on system::*fadE*::T*trpC*, *pyrG*^+^	Overexpression of *fadE*	This study
OE-*fadA*	YS20.2, Tet-on system::*fadA*::T*trpC*, *pyrG*^+^	Overexpression of *fadA*	This study
OE-*hyp*	YS20.2, Tet-on system::*hyp*::T*trpC*, *pyrG*^+^	Overexpression of *hyp*	This study
OE-cyp	YS20.2, Tet-on system::c*yp*::T*trpC*, *pyrG*^+^	Overexpression of c*yp*	This study
OE-acy	YS20.2, Tet-on system::*acy*::T*trpC*, *pyrG*^+^	Overexpression of *acy*	This study
Plasmids			
pFW22.1	Recombinant plasmid containing Tet-on system	For subsequent plasmid construction	[16]
pOE03	Tet-on system::*fadE*::T*trpC*, pyrG^*^	For construction of strain OE-*fadE*	This study
pOE057	Tet-on system::*fadA*::T*trpC*, pyrG^*^	For construction of strain OE-*fadE*	This study
pOE058	Tet-on system::*hyp*::T*trpC*, pyrG^*^	For construction of strain OE-*fadE*	This study
pOE06	Tet-on system::c*yp*::T*trpC*, pyrG^*^	For construction of strain OE-*fadE*	This study
pOE08	Tet-on system::*acy*::T*trpC*, pyrG^*^	For construction of strain OE-*fadE*	This study

### Construction of plasmids and overexpression strains

Integrative plasmid pFW22.1 [16] contains doxycycline-inducible Tet-on system and unfunctional orotidine-5′-phosphate decarboxylase (pyrG^*^) fragment, which contributes to site-specific integration of overexpression cassettes into *A. niger* genome. Five candidate genes amplified from B36 genome by primers F-03240/R-03240, F-05720/R-05720, F-05840/R-05840, F-06460/R-06460 and F-08870/R-08870 were cloned at the *Pme*I site on pFW22.1 to generate overexpression plasmids pOE03, pOE057, pOE058, pOE06 and pOE08. Then these plasmids were transformed in parental strain YS20.2 to integrate into the *pyrG* site of genome by homologous recombination. And the transformation of *A. niger* were performed as previously described in Arentshorst et al. [17]. All transformants were screened by uridine prototrophy. All strains and plasmids used in this study are listed in Table 1, and primers are listed in Additional file 1: Table S1.

### Medium

Flask-level fermentation medium contained (g/L): Maltose 30, Tryptone 10, Yeast extract 5, KH_2_PO_4_ 1, MgSO_4_·7H_2_O 0.5, FeSO_4_·7H_2_O 0.3, ZnCl_2_ 0.03, CaCl_2_ 0.02, MnSO_4_·7H_2_O 0.01, Tween 80 3 mL, and pH was adjusted to 5.5 by 1 M HCl. Maltose-limited medium in initial state of chemostat culture contained (g/L): NH_4_Cl 4.5, KH_2_PO_4_ 1.5, KCl 0.5, MgSO_4_·7H_2_O 0.5, Yeast extract 0.03, 1000 × Trace element (g/L: EDTA-2Na 10, ZnSO_4_·7H_2_O 4.4, MnCl_2_·4H_2_O 1.01, CoCl_2_·6H_2_O 0.32, CuSO_4_·5H_2_O 0.315, (NH_4_)_6_Mo_7_O_24_·4H_2_O 0.22, CaCl_2_ 1.11, FeSO_4_·7H_2_O 1) 1 mL, Defoam (Polypropylene glycol) 10, and pH was adjusted to 3. Maltose was separately sterilized and added at 10 g/L final concentration. The composition of feed medium in chemostat culture was as same as the initial medium except yeast extract.

### Shake-flask cultivation

To evaluate the glucoamylase production performance of engineered strains, 10^6^ spores/mL of reference strain B36 and them were inoculated into 250-mL flask containing 50 mL medium and cultured at 30 °C with 250 rpm for 90 h. 20 µg/mL doxycycline (DOX) was added as inducer at 18 and 42 h cultivation, respectively, and supplemented with same dose every 12 h after initial induction. Samples were taken at 18 h, 54 h, 66 h, 78 and 90 h for measurement of dry cell weight. And glucoamylase activity and total secreted protein were determined at the end of cultivation. Experiments were performed in biological quadruplicates.

### Chemostat cultivation

10^9^ spores/L of conidial suspension was inoculated into 5 L bioreactor (NCBIO, Shanghai, China) containing 3 L medium and cultured with an agitation of 250 rpm and a constant air flow of 1 VVM for 2 h to avoid loss of conidia. After 2 h, the agitation and aeration were lifted to 750 rpm and 2 vvm, respectively. During whole cultivation, the pressure and temperature were maintained at 0.05 MPa and 30 °C, individually, and pH was controlled at 3 by 2 M NaOH or 1 M HCl. The cultivation switched from batch to chemostat in late-logarithmic growth phase when OUR (Oxygen Uptake Rate) and CER (Carbon-dioxide Evolution Rate) started to decline. The dilution (D) rate was set at 0.1 h^− 1^ by controlling the feed flow. The steady-state defined by constant OUR, CER and biomass concentration was reached in about 4–5 elution volumes. To monitor cell growth and determine whether steady-state had been reached, samples were taken regularly from 12 h. The sampling interval was 2 h during batch cultivation and extended to 10 h during chemostat cultivation. When biomass concentration reached 1–2 g dry weight per kg of culture, 10 µg/mL DOX was added. And DOX was also applied to feeding medium at same dose. Samples were taken at steady-state to quantify intracellular metabolites and free fatty acids. Total secreted protein and glucoamylase content were determined after fermentation.

### Determination of dry cell weight, total secreted protein, glucoamylase content and activity

Biomass was harvested by pre-weighed filter paper from a known mass of culture broth (3–4 mL samples at the indicated time points from shake flask or bioreactor), washed twice by deionized water and dried in oven for 2 days to determine dry cell weight (DCW). The dry cell weight was defined as dried total weight minus filter paper weight and then divided by the weight of culture broth. Total secreted protein in broth supernatant was determined via BCA Protein Assay Kit (Beyotime, Shanghai, China) with bovine serum albumin (BSA) as a standard. The content of glucoamylase was measured by an enzyme-linked immunoassay kit (Yancheng biotechnology, Shanghai, China).

Enzyme activity of glucoamylase was measured by a standard procedure [18]. The supernatant was obtained from 4 mL culture broth by centrifugation. And 230 µL p-nitrophenyl-α-D-glucopyranoside (prewarmed at 37 °C for 5 min) substrate was mixed with a 20 µL obtained supernatant sample (diluted as needed) and incubated at 37 °C for 20 min. Then the reaction was terminated by adding 100 µL Na_2_CO_3_ (3 mol/L). The absorption was measured at 405 nm by Varioskan LUX multimode microplate reader (Thermo, Waltham, USA). A control or standard sample was taken along in the experiment to be able to determine the absolute enzyme activity of glucoamylase. The enzyme activity of glucoamylase was expressed in AGI units (Amyloglucosidase). One AGI unit was defined as the amount of enzyme that produces 1 µmol of glucose per minute at pH 4.3 and at 60 °C from a soluble starch substrate.

### Sampling and quantification of intracellular metabolites

The sampling and quantification of intracellular metabolites was slightly modified based on Wang et al. [19]. 1–2 mL culture broth (precisely weighed) was transferred by a rapid sampling protocol from bioreactor into 10 mL precooled quench solution (60% v/v methanol at − 30 °C). Isotope dilution mass spectrometry (IDMS) [20] was used in this work for the quantification of metabolite concentrations. After quickly filtered and rinsed with 20 ml pre-cooled quenching solution, the remaining cells with filter paper and 100 µL ^13^C internal standard solution was added to 25 mL pre-warmed ethanol (75% v/v, 75 °C) for 3 min at 95 °C to extraction intracellular metabolites. After cooling and filtering again to remove cell debris, the obtained metabolite extract was concentrated to 600 µL by rotary evaporation and stored at − 80 °C for subsequent measurement.

At the quantification of amino acids in cells, 100 µl samples were taken out and dried in a freeze dryer overnight. Left dry crystals was dissolved in 75 µl acetonitrile and at 70 °C for 1 h. Then 75 µl N-methyl-N-(tert-butyldimethylsilyl) trifluoroacetamide was added and the solution was derived at 70 ° C for 1 h. The derivatized solution was then centrifuged (12,000 rpm, 1 min) and 100 µl supernatant transferred to a sample vial for GC/MS analysis (7890 A & 5975 C; Agilent Technologies Inc.) equipped with non-polar elastic quartz capillary column (HP-5MS, 30 m × 0.25 mm×0.25 μm). In the process of material analysis, the selective ion monitoring (SIM) mode is used to accurately quantify a single amino acid, and the scanning range of mass spectrometry is 1-1050 amu [21]. The detection parameters are set as follows: the carrier gas velocity of high-purity helium is 1 ml / min, the sample injection volume is 1 µL, the ion source temperature is controlled at 230 °C, the quadrupole temperature is controlled at 150 °C, and the transmission line temperature is controlled at 250 °C.

Concentrations of other kind of central carbon metabolism were quantified by LC-MS/MS (Thermo Fisher Scientific, San Jose, USA) and the analysis method is set as follows: The instrument contains a vanguard precolumn, 1.7 μm, 2.1 mm×Acquity UPLC beh C18 column, 1.7 μm, 2.1 mm×150 mm), and the column temperature is controlled at 25  °C. According to the performance of the instrument, in the detection system, the mobile phase is divided into two parts: acetonitrile containing 5% 5 mmol/L dibutylammonium acetate (DBAA) and acetonitrile containing 84% 5 mmol/L DBAA. The flow rate of the mobile phase is 0.2 mL/min. When detecting substances, only 5% acetonitrile containing 5 mmol/L DBAA in the first 0–20 min, and then 20% acetonitrile containing 84% 5 mmol/L DBAA for 2 min; Then, add 100% acetonitrile containing 5 mmol/L DBAA for 10 min until the end of the test. The injection volume of each sample is 2 µL. Hundred-microliter concentrated extract was filtered using 0.22 μm Nylon filter and then transferred to a sample vial for LC-MS/MS analysis.

In order to obtain the standard curve of IDMS IS (^13^C internal standard), mixture of ^13^C labeled cell extract (all carbon atoms are ^13^C isotopes) and ^12^C metabolites (with a series of standard concentration) from Sigma with volume ratio of 1:5 was injected to the MS/MS system. Then the peak height ratio of ^13^C and ^12^C metabolites was regressed with the concentration of ^12^C metabolites to obtain the standard curve.

### Quantification of intracellular free fatty acids

The mycelium was obtained from 1 to 2 mL quenched broth through centrifugation at 12,000 rpm for 5 min and resuspended to 1 mL extraction solution. The above suspension with 500 µL micro-beads was disrupted at 65 Hz for 3 min in grinder (Jingxin, Shanghai, China) and vortexed for 3 h. After centrifugation at 4 °C 8,000 rpm for 10 min, the supernatant was collected to quantify free fatty acids (FA) by FFA Assay Kit (Solarbio, Beijing, China).

### Quantification of extracellular oxalic acid

Samples taken regularly from bioreactor fermentation were filtrated by 0.22 μm membrane. The main extracellular by-product oxalic acid was quantified by HPLC (Shimadzu, Kyoto, Japan). 5 mM H_2_SO_4_ was used to wash VARIAN Metacarb H plus column at a flow rate of 0.4 mL/min at 50 °C, and the acid was detected at a wavelength of 210 nm.

### Determination of gene copy number and expression level of target genes

The quantification methods of gene copy number and RNA level were adapted from Xiao et al. [19]. To determine copy number of target genes in positive transformants, the genome DNA was extracted as described in Arentshorst et al. [17]. 1 µL appropriately diluted genome template was mixed with 2 × SYBR Green Pro HS Premix (Accurate Biology, Changsha, China), corresponding primers and ddH_2_O for preparation of real-time quantitative PCR (RT-qPCR) reaction systems. The housekeeping gene glyceraldehyde-3-phosphate dehydrogenase (*gapdh*) was selected as internal standard. The qPCR was performed in CFX96 (Bio-Rad, Hercules, USA) as follows: 95 °C for 30 s, then 40 cycles of 95 °C for 5 s and 58 °C for 30 s.

2 mL fermentation broth was taken from bioreactor at mid-logarithmic growth phase and steady-state. The mycelium was harvested by centrifugation (13,000 rpm for 2 min), washed by DEPC-H_2_O and frozen in liquid nitrogen immediately. After liquid nitrogen grinding, RNA was obtained via Fungal Total RNA Isolation Kit (Sangon, Shanghai, China). 1 µg total RNA was applied for cDNA synthesis using *Evo M-MLV* RT Kit (Accurate Biology) according to manufacturer’s instructions. Subsequent RT-qPCR was performed as same above reaction systems and conditions. All the primers used in this part are listed in Additional file 1: Table S1.

### Central carbon metabolic flux estimation

Genome scale metabolic network model (GSMM) IHL1210 [23] was used to estimate central carbon metabolic flux in steady-state. Maximum cell growth was selected as the objective of the optimization function with the input constraints including the measured values of qS, qO_2_ and qCO_2_, etc. According to previous experience, in order to ensure the stability of the model and the normal flux of maltose utilization pathway, the mutual conversion reaction between NADH and NADPH (R1289 and R1290), the absorption of urea and nitric acid and the transmembrane transport of malic acid and citric acid (R1584 and R1585) were closed before estimating the metabolic flow, and the conversion reaction from FAD to FADH2 was closed to eliminate the growth of cells without carbon source input. These reactions are closed by setting the upper and lower limits of the above flux to 0 in advance. All metabolic flux estimating work was carried out on MATLAB with cobra toolbox [24] and Gorubi (Version 9.0.1) was used as the solver. The metabolic reaction formula and corresponding metabolites mentioned in the following analysis are listed in Additional file 1: Tables S2 and S3.

## Results

### Construction of mutants overexpressing FA degradation pathway genes

Based on our previous transcriptome analysis of oxygen-limited fermentation for glucoamylase production in *A. niger* [13], 5 genes, related to fatty acids degradation and continuously up-regulated during oxygen-limitation period, were selected as candidate genes (Table 2). Five genes named *fadE* (encoding Acyl-CoA dehydrogenase), *fadA* (encoding Acetyl-CoA C-acyltransferase), *hyp* (encoding hypothetical protein with hydrolase activity), *cyp* (encoding cytochrome P450 monooxygenase), *acy* (encoding acyltransferases) were overexpressed in the *pyrG* locus of *A. niger* YS20.2 genome under the control of Tet-on system (Table 1). The positive mutants with single-copy gene integration (besides endogenous one) were verified by colony PCR and quantitative PCR (Additional file 1: Table S4).

**Table 2:** Candidate genes related to fatty acids degradation in *A. niger*

Protein ID^*^ of gene product in *A. niger* strain		Annotation	EC number	Gene
CBS513.88	N402			
An14g03240	ATCC64974_2860	Acyl-CoA dehydrogenase	1.3.99.-	*fadE*
An04g05720	ATCC64974_80890	Acetyl-CoA C-acyltransferase	2.3.1.16	*fadA*
An04g05840	ATCC64974_80990	Hypothetical protein (hydrolase activity, acting on ester bonds activity and role in lipid metabolic process)	2.3.1.-	*hyp*
An03g06460	ATCC64974_75860	Cytochrome P450 monooxygenase	1.14.-.-	*cyp*
An08g08870	ATCC64974_99590	Acyltransferases	2.3.-.-	*acy*

*Protein ID number for both strains are from the FungiDB database (https://fungidb.org/)

### Properties of engineered strains on flask-level cultivation

**Fig. 1:**
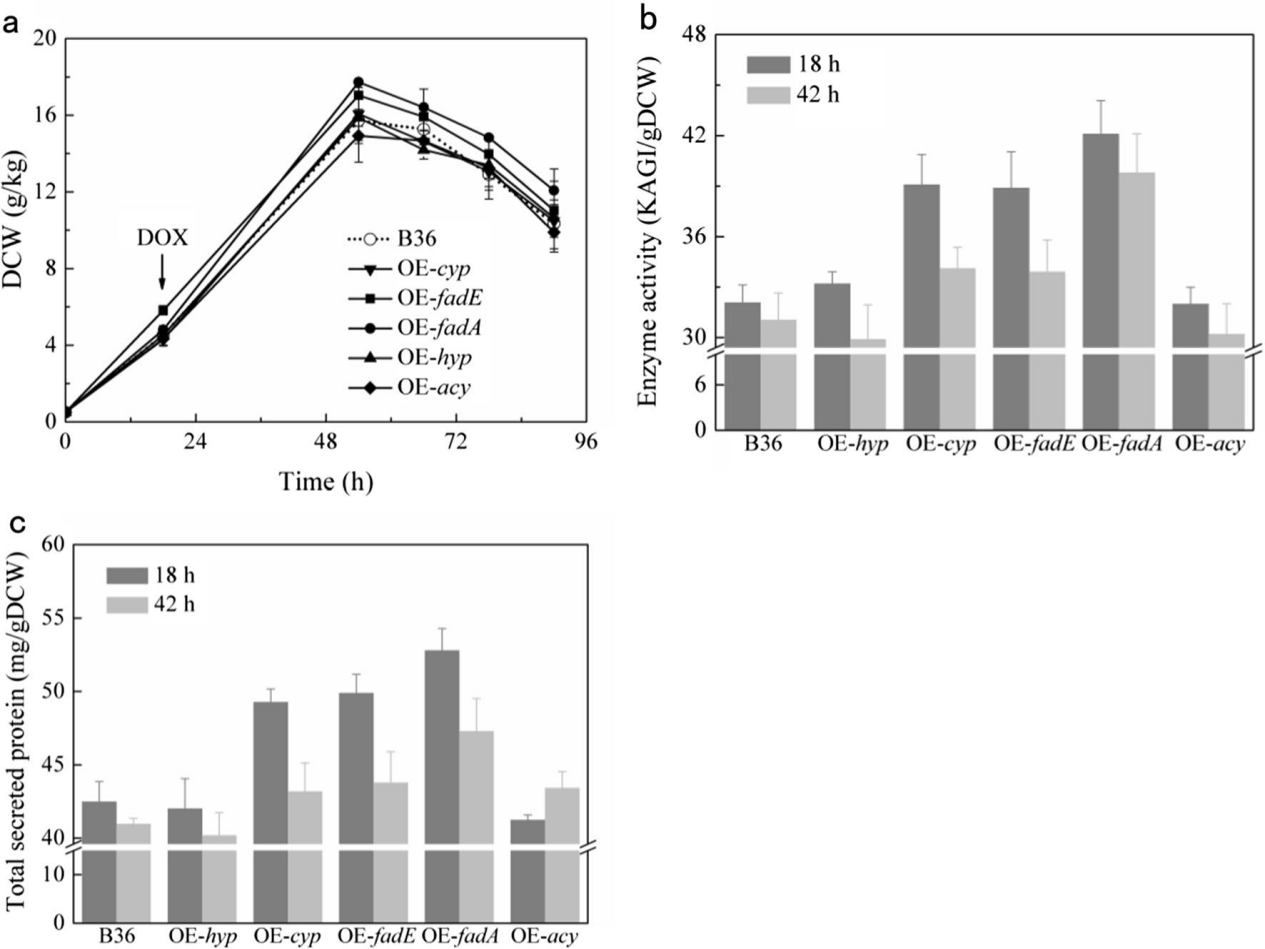
Properties of overexpression strains on flask-level cultivation. **a**, Biomass accumulation of all strains with supplement of inducer DOX since 18 h. **b**, **c**, Glucoamylase activity and total secreted protein of all strains with the supplement of inducer since either 18 or 42 h. DCW, dry cell weight. DOX, doxycycline. Genes *hyp*, *cyp*, *fadE*, *fadA* and *acy* were overexpressed in strains OE-*hyp*, OE-*cyp*, OE-*fadE*, OE-*fadA* and OE-*acy.* Error bars represent the standard errors of average values from three biological replicates

In order to screen out the strains with the ability of promoting glucoamylase synthesis, 5 overexpression mutants were all cultivated in shake flask. The dry cell weight of mutants OE-*fadE* (overexpressing *fadE* gene) and OE-*fadA* (overexpressing *fadA* gene) were accumulated slightly compared to reference strain B36 and others during this cultivation (Fig. 1a). When target genes were continuously induced by DOX since early exponential growth phase (18 h), the final specific glucoamylase enzyme activities of strain OE-*fadA*, OE-*fadE* and OE-*cyp* (overexpressing *cyp* gene) were increased by 31.3% (42.1 KAGI/gDCW), 21.3% (38.9 KAGI/gDCW) and 21.9% (39.1 KAGI/gDCW) respectively compared with B36 (32.08 KAGI/gDCW) (Fig. 1b). And the final total secreted protein levels of strain OE-*fadA*, OE-*fadE* and OE-*cyp* were 24.2%, 17.4% and 16.0% higher than B36, respectively (Fig. 1d). When DOX was supplemented since early stationary phase (42 h), overexpression of genes *fadE*, *fadA* and *cyp* also improved enzyme activity and secretion of protein, but not as effective as supplementation since 18 h. However, the mutants OE-*hyp* (overexpressing *hyp* gene) and OE-*acy* (overexpressing *acy* gene) with supplement of DOX since either 18 or 42 h showed no difference with B36 in specific enzyme activity and total secreted protein (Fig. 1b, c).

### Performance of selected strains on chemostat culture

**Fig. 2:**
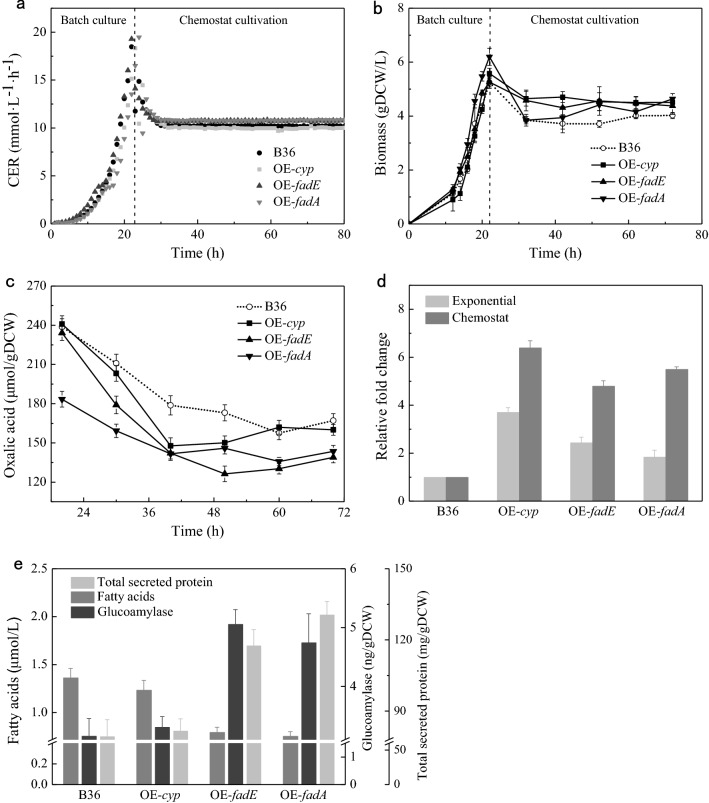
Performance of selected strains on chemostat fermentation. **a** CO_2_ evolution rate (CER) in mmol·L^− 1^·h^− 1^ during whole fermentation. **b** Growth curves of selected strains OE-*cyp* (overexpressing *cyp*), OE-*fadE* (overexpressing *fadE*), OE-*fadA* (overexpressing *fadA*) and B36. **c** By-product oxalic acid of all strains. **d** Relative fold change of genes *fadE*, *fadA* and *cyp* at mid-exponential growth phase and steady state. The expression levels of 3 genes in overexpression strains were normalized by their values, set to 1, in B36. **e** Intracellular free fatty acids, extracellular glucoamylase and total secreted protein of all strains at steady state. Error bars represent the standard errors of the means of three biological replicates

Based on flask-level results, the strains OE-*fadA*, OE-*fadE* and OE-*cyp* were selected to inoculate in 5 L bioreactor for maltose-limited chemostat fermentation. The effects of gene overexpression on the global metabolism were expected to be further compared by the chemostat culture experiment. In the course of the fermentation, the inducer DOX was supplemented since early exponential growth phase (12 h). After about 22 h, the cultivation switched from batch to chemostat (Fig. 2a). The results showed that 3 overexpression strains exhibited a little higher biomass but lower oxalic acids than reference strain B36 during the batch and chemostat phases (Fig. 2b and c), which was also reflected by calculated physiology parameters (Table 3). Transcript levels of genes *fadA*, *fadE* and *cyp*, induced by DOX, were 1.84-, 2.44- and 3.71-fold higher in mid-exponential growth phase and 5.52-, 4.83- and 6.41-fold higher in steady-state than that of B36 (Fig. 2d). As expected, overexpression of *fadA*, *fadE* and *cyp* reduced intracellular free FA content in corresponding strains by 44.6%, 41.6% and 9.4%, individually in chemostat phase (Fig. 2e). The final extracellular glucoamylase content and total secreted protein of OE-*fadA*, OE-*fadE* and OE-*cyp* were increased by 31.2% (4.75 ng/gDCW), 34.1% (5.06 ng/gDCW), 6% (3.31 ng/gDCW) and 38.8% (130.56 mg/gDCW), 35.1% (117.55 mg/gDCW), 3% (81.66 mg/gDCW) relative to B36 (3.16 ng/gDCW, 79.30 mg/gDCW), respectively (Fig. 2e). Therefore, the enhanced expression of these three genes all promoted intracellular fatty acids degradation, thereby improving protein secretion and glucoamylase synthesis, especially genes *fadA* and *fadE*.

**Table 3:** Physiology parameters of different strains in chemostat culture

	B36	OE-*cyp*	OE-*fadE*	OE-*fadA*
µ (h^− 1^)	0.09 ± 0.01	0.09 ± 0.02	0.09 ± 0.01	0.09 ± 0.01
C_Biomass_ (g_DCW_/kg)	3.91 ± 0.12	4.52 ± 0.2	4.47 ± 0.36	4.4 ± 0.21
q_CO2_ (mmol/g_DCW_·h)	2.61 ± 0.03	2.21 ± 0.06	2.40 ± 0.07	2.45 ± 0.04
q_O2_ (mmol/g_DCW_·h)	2.47 ± 0.07	2.10 ± 0.09	2.21 ± 0.10	2.24 ± 0.10
q_s_ (mmol_maltose_/g_DCW_·h)	0.75 ± 0.02	0.65 ± 0.03	0.65 ± 0.01	0.66 ± 0.02
RQ	1.06 ± 0.05	1.05 ± 0.08	1.09 ± 0.09	1.09 ± 0.07
Carbon recovery	95%	102%	105%	104%

*C*_*biomass*_ biomass concentration (dry cell weight (DCW)),* q*_*CO2*_ specific carbon dioxide evolution rate,* q*_*O2*_ specific oxygen uptake rate,* RQ* respiratory quotient calculated as the ratio of CO_2_ production and O_2_ consumption rates. q_s_, specific substrate consumption rate. Standard deviations (±) from duplicate independent steady-state results

### Quantification of intracellular amino acids and metabolites of mutants in chemostat culture

**Fig. 3:**
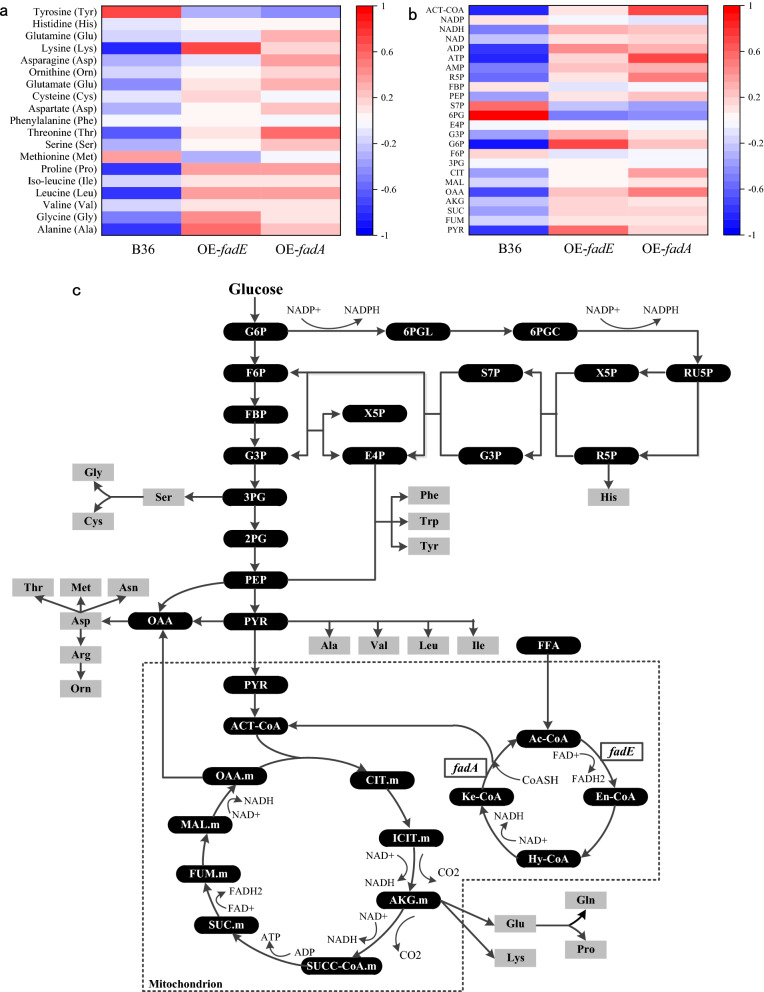
Metabolic profiling of amino acid pools and central carbon metabolism in 2 overexpression strains and reference strain B36 at steady state. **a** and **b** The relative pool size of amino acids and intracellular metabolites in OE-*fadE* (overexpressing gene *fadE*), OE-*fadA* (overexpressing gene *fadE*) and B36. The average content of each metabolite (C_mean_) in the three strains was set as the reference and the relative content was calculated as (C-C_mean_)/C_mean_. **c** Fatty acids degradation (β-oxidation), amino acid synthesis and center carbon metabolism pathway

According to the amino acids (AAs) quantification, the total AA pools of OE-*fadA* and OE-*fadE* were increased by 18.49% and 24.86% at steady state compared to B36, respectively. Except for 2 kinds of decreased AAs (tyrosine, methionine), 3 kinds of unchanged AAs (phenylalanine, cysteine and histidine), other 14 out of 19 kinds of intracellular AAs were boost in the 2 overexpression strains, especially those AAs that could be used as precursors for other AAs synthesis such as glutamic acid, aspartic acid and serine (Fig. 3a, Additional file 1: Table S5). The 5 most abundant AAs in *A. niger* glucoamylase composition are serine, threonine, alanine, leucine, glycine. And alanine, glutamate, glycine, leucine, lysine are the 5 most abundant AAs in cell biomass. Combining together, there are 7 kinds of AAs abundant in either cellular or enzymatic proteins. As predicted, the intracellular pools of these common 7 AAs were remarkably higher in 2 overexpression strains than that in reference strain. Notably, the serine and threonine pools in OE-*fadE* were higher than those in OE-*fadA*, which explained the higher glucoamylase production and lower total secreted protein in the former.

The results of central carbon metabolites quantification suggested that overexpressing *fadA* and *fadE* genes increased metabolite pools of tricarboxylic acid (TCA) cycle with varying degrees compared to B36, especially the acetyl-CoA abundance (Fig. 3b). The carbon flux was redirected to glyceraldehyde-3-phosphate (G3P) through pentose phosphate pathway (PPP) in OE-*fadA* and OE-*fadE*, which reduced fructose-6 -phosphate (F6P) and 6-phosphogluconic acid (6PG) pools. The accumulation of phosphoenolpyruvate (PEP), pyruvate (PYR) and α-ketoglutaric acid (AKG) pools in 2 overexpression strains was supposed to provide more precursors for aromatic, pyruvate-derived and glutamate amino acids synthesis. In addition, 5 metabolites (G6P, G3P, R5P, PEP and PYR) representing EMP and PPP were also increased in the 2 engineered mutants compared to B36. Up-regulated ATP pools feedback inhibited the activity of phosphofructokinase involved in the transformation from G6P to F6P, resulting in a reduction of F6P pool [20]. Higher ATP and NADH pools enabled driving force for AAs biosynthesis, while increased AMP, ADP and NAD^+^ pools contributed to maintain intracellular redox balance [21].

### Central carbon metabolism flux estimation of mutants in chemostat culture

The relative fluxes of central carbon metabolism in OE-*fadA*, OE-*fadE* and B36 under the chemostat culture were simulated through the *A. niger* genome-scale metabolic network model iHL1210. The predicted results (Fig. 3c and Additional file 2: Fig. S1) indicated that the flux of glycolytic pathway (EMP) was slightly improved, flux of PPP mildly diminished and flux of TCA cycle apparently boost in 2 overexpression compared to B36. Notably, the flux of TCA cycle was increased more in OE-*fadA* than in OE-*fadE*. In general, the determination results of amino acids, organic acids, sugar phosphates and energy substances were consistent with metabolic flux analysis results. These data revealed that enhancement of fatty acids degradation significantly raised intracellular supply of acetyl CoA, NADH and ATP, then increased metabolic fluxes to TCA cycle to improve amino acids synthesis derived from TCA intermediates, finally further promoted enzyme and other protein production.

## Discussion

Fatty acids are main components of cell lipids in cell membrane and participate in many important physiological activities such as anabolism, energy metabolism and signal transduction in organisms [22]. During cell growth, a lot of intracellular free fatty acids (FFA) gradually accumulate. Thus, accelerating FFAs degradation was supposed to provide more sufficient precursors, energy and reducing power for amino acids and protein synthesis. At present, three fatty acids degradation pathways have been reported in eukaryotes and prokaryotes including α-, β- and ω-oxidation, among which β-oxidation is the major degradation pathway [23].

In this study, overexpression of *fadE* and *fadA* genes, involved in β-oxidation, exhibited significantly positive effect on glucoamylase production during batch and chemostat cultivation. Thereinto, the acyl-CoA dehydrogenase, encoded by gene *fadE*, is an electron-transferring flavoprotein containing FAD cofactor and catalyzes the dehydrogenation of acyl-CoA to form a C = C double bond in β-oxidation [24]. Enhancing *fadE* gene expression might increase dehydrogenation of acyl-CoA, resulting in the highest reducing power of OE-*fadE* in 5 overexpression strains. Since the abundance of the total amino acids pool is positively correlated with the intracellular reducing power, this may contribute for the highest total AAs pool of OE-*fadE*. The acetyl-CoA C-acyltransferase, encoded by *fadA*, is responsible for the last thiolysis reaction in β-oxidation to yield acetyl-CoA and an acyl-CoA that is chain-shortened by 2 carbon atoms [25]. Acetyl-CoA can be further catalyzed by citrate synthase together with oxaloacetate to generate citric acid into TCA cycle, and acyl-CoA was directly into next cycle of β-oxidation. Therefore, the efficiency of fatty acids β-oxidation in OE-*fadA* might be higher than in OE-*fadE*, which corresponds to the results of higher TCA flux, more intracellular ATP and acetyl-CoA and less FFAs in OE-*fadA*. In sum, elevation of CER in OE-*fadE* and OE-*fadA* revealed an enhancement of the fatty acid degradation releasing more acetyl-CoA into the TCA cycle (Fig. 2b). And higher intracellular cytosolic oxaloacetate (OAA) and lower extracellular oxalic acid also indicated that more oxaloacetate flowed into the TCA cycle and aspartate amino acid synthesis instead of oxalic acid production in these two overexpression strains than B36, enabling more ATP, NADH and amino acid precursors for protein production (Fig. 2g).

In addition, the overexpression of gene *cyp*, involved in ω-oxidation, also exhibited slightly positive effect on glucoamylase synthesis. Hydroxylation of FFAs was catalyzed by cytochrome P450 monooxygenase (encoded by *cyp* gene) to form ω-hydroxy fatty acids in ω-oxidation [26]. The final α, ω-dicarboxylic acid produced by ω-oxidation may enter into β-oxidation for further degradation. However, ω-oxidation, usually as minor fatty acid oxidation pathway, is up-regulated to compensate blocked β-oxidation pathway [27]. While the flask-level results were not totally consistent with the bioreactor-level, the bioreactor-level determinations of genetic modification effects on cell physiology and metabolism are more reliable due to its constant specific growth rate and stable fermentation condition (dissolved oxygen, pH etc.). It is worth mentioning that FFAs and ω-hydroxy FAs are both quantified as total FFAs by assay kit, which may explain the little increasement of intracellular FFAs in OE-*cyp* compared to B36.

Overexpression of *hyp* and *acy* genes had no obvious effect on glucoamylase biosynthesis. The *hyp* gene was predicted as a hypothetical protein with hydrolase activity, acting in the late phase of FA β-oxidation process. The limited effect of overexpression of *hyp* may due to its less impact on late β-oxidation process. The acylation reaction catalyzed by acyltransferase (encoded by *acy*) is widely involved in many physiological activities in cells [28], and overexpression of this gene seems to be harmful to cell growth and enzyme production (Fig. 1b and c).

In summary, the reasons for glucoamylase production increase in OE-*fadE* and OE-*fadA* might be manifold. On the one hand, enhancement of fatty acids degradation significantly promoted most amino acids pools and the precursor amino pools of glucoamylase, such as alanine, glycine and leucine. In a recent study, improving cytosolic aspartate synthetic pathway exhibited significantly increased glucoamylase activity by 23.5% and 60.3% compared to parental strains on shake-flask and 5 L-bioreactor level, respectively [11]. On the other hand, enhancement of fatty acids degradation also contributed to higher ATP and NADH pools for protein synthesis. Energy and cofactor supply could be bottlenecks for improving glucoamylase in high yield strain of *A. niger*. For example, overexpressing *gndA* (encoded 6-phosphogluconate dehydrogenase) or *maeA* (encoded NADP-dependent malic enzyme) gene involved in NADPH supply pathway also increased the yield of glucoamylase by 65% and 30%, respectively [12]. In addition, independently overexpressing 3 NADH kinases (*AN03*, *AN14*, *AN17*) and malic enzyme (*maeA*) improved glucoamylase activity by 70%, 50%, 90% and 70%, respectively. The combination of genes *AN17* (encoded mitochondrial NADH kinase) and *maeA* increased enzyme activity by further 19% [29]. In previous study, Lu et al. found the imbalance in NADH regeneration and consumption and the low availability of NADPH may limit the overproduction of glucoamylase [21]. Therefore, the systematic metabolic engineering strategy could effectively improve rational strain development of cell factories and deepen the understanding of resources allocation in cell factories.

## Conclusion

Encouraged by our data, overexpression of 2 genes involved in fatty acids β-oxidation accelerated intracellular fatty acids degradation and significantly promoted glucoamylase biosynthesis. The global mechanism was revealed by metabolomic study under chemostat culture as enhancement of FFAs degradation provided more precursors, energy and reducing power to TCA cycle and derived AAs synthesis, and finally increased enzyme production. Therefore, this strategy could be broadly applied to rational optimization of enzyme production in other microbial cell factories.

## Supplementary Information

**Additional file 1: Supplementary tables. Table S1**. Primers used in this study.** Table S2**. Metabolic reaction formula of relative flux simulation of central carbon metabolism.** Table S3**. Metabolite abbreviations and descriptions mentioned in formula.** Table S4**. Copy number of target genes in overexpression strains identified by quantitative PCR. **Table S5**. Pool sizes of intracellular amino acids for strains OE-*fadE* (overexpressing gene *fadE*), OE-*fadA* (overexpressing *fadA*) and B36 at steady state. The unit is μmol/gDCW.

**Additional file 2: Supplementary figures. Fig. S1**. The relative flux of EMP, PP and TCA pathways in control strain B36 and two recombinant strains at chemostat culture steady state predicted by iHL1210.

## Data Availability

All data generated or analyzed during this study are included in this article and its supplementary information files.

## Abbreviations

G6P: Glucose-6-phosphate
F6P: Fructose-6-phosphate
FBP: 1,6 Fructose diphosphate
G3P: Glyceraldehyde-3-phosphate
3PG: 3-Phosphoglycerate
2PG: 2-Phospho-D-glycerate
PEP: Phosphoenolpyruvate
PYR: Pyruvate
OAA: Cytosolic oxaloacetate
ACT-CoA: Acetyl-CoA
CIT.m: Mitochondrial citrate
ICIT.m: Mitochondrial isocitrate
AKG.m: Mitochondrial α-ketoglutaric acid
SUCC-CoA.m: Mitochondrial succinyl-CoA
SUC.m: Mitochondrial succinate
FUM.m: Mitochondrial fumarate
MAL.m: Mitochondrial malate
OAA.m: Mitochondrial cytosolic oxaloacetate
6PGL: D-glucono-1,5-lactone 6-phosphate
6PGC: 6-Phospho-D-gluconate
RU5P: Ribulose-5-phosphate
X5P: D-xylulose-5-phosphate
R5P: Ribose-5-phosphate
S7P: Sedoheptulose-7-phosphate
G3P: Glyceraldehyde-3-phosphate
E4P: Erythrose-4-phosphate
FFA: Free fatty acids
Ac-CoA: Acyl-CoA
En-CoA: *trans*-Δ^2^-Enoyl-CoA
Hy-CoA: 3-L-hydroxyacyl-CoA
Ke-CoA: β-Ketoacyl-CoA
CoASH: Coenzyme A
