# Supplementary material for: Enhancement of fatty acid degradation pathway promoted glucoamylase synthesis in Aspergillus niger
Source: Microb Cell Fact. 2022 Nov 15;21:238. doi: 10.1186/s12934-022-01966-3 (PMC9664828; doi:10.1186/s12934-022-01966-3)
Supplement: Supplementary file 1 — Additional file 1: Supplementary tables. Table S1. Primers used in this study. Table S2. Metabolic reaction formula of relative flux simulation of central carbon metabolism. Table S3. Metabolite abbreviations and descriptions mentioned in formula. Table S4. Copy number of target genes in overexpression strains identified by quantitative PCR. Table S5. Pool sizes of intracellular amino acids for strains OE-fadE (overexpressing gene fadE), OE-fadA (overexpressing fadA) and B36 at steady state. The unit is μmol/gDCW. [file 12934_2022_1966_MOESM1_ESM.docx]

Table S1 Primers used in this study.

| Primer | Nucleotide sequence (5’to3’)^*^ |
| --- | --- |
| Plasmids Construction | |
| F-fadE | cagacatcaccgtttatgTCCCGTATATACCAACTAAGC |
| R-fadE | cggcatctactgttttTAATAACTCCTCGGCAACTGC |
| F-fadA | cagacatcaccgtttatgTCTTCCGGTAAGTCTACAG |
| R-fadA | cggcatctactgttttTACTCCGCAACCCAAACG |
| F-hyp | cagacatcaccgtttatgCGCCTCTTTAGCAAATTT |
| R-hyp | cggcatctactgttttCATCGGAGCCACATCTCTT |
| F-cyp | cagacatcaccgtttatgTTAGGACTCTGGTGTGC |
| R-cyp | cggcatctactgttttCATATCTCACGGGGAGTGAA |
| F-acy | cagacatcaccgtttatgCCCAAAACATGCATTCG |
| R-acy | cggcatctactgttttTATAAAAACTTTGCTTCCCGCAC |
| RT-qPCR | |
| RT-fadE-F | AGCTGATTATGCGAAGGAGAG |
| RT-fadE-R | CCGCCTCCAACCTCATATAC |
| RT-fadA-F | ATTGCCAATGCCCTGAAATC |
| RT-fadA-R | GGTGAAGGGAGCAGAAACTTA |
| RT-hyp-F | CGCAGATTACCAAGCACAAAG |
| RT-hyp-R | CAAATAGATCGTGCCGTTTGAC |
| RT-cyp-F | CTGATGGAGGAATTAACGTACCA |
| RT-cyp-R | TGGCAGGTAGTAAGGAAGTAGA |
| RT-acy-F | ACACCAGAAGCCTCACAAATA |
| RT-acy-R | GACTGAACGGGTCAGAGAAAG |
| qGAPDHF | CTCCTACACCAAGGACATCAAC |
| qGAPDHR | TCAACGATGCCGAACTTGT |
| Colony PCR | |
| jpVF | GTCTCCGCAAGGAAGCTGAT |
| jpVR | GGGGCGAAAACTCTCAAGGA |

* Non-specific sequences are indicated in lowercase letters.

Table S2 Metabolic reaction formula of relative flux simulation of central carbon metabolism.

| Description | Reaction |
| --- | --- |
| α-Glucosidase (Maltase) | h2o[C_c] + mlt[C_c] =>2 glc [C_c] |
| Aldose 1-epimerase | glc[C_c] <=> bdglc[C_c] |
| Hexokinase (hxk) | glc[C_c] + atp[C_c] => adp[C_c] + g6p[C_c] + h[C_c] |
| Glucose-6-phosphate isomerase | g6p[C_c] <=> f6p[C_c] |
| 6-phosphofructokinase | atp[C_c] + f6p[C_c] => adp[C_c] + h[C_c] + fdp[C_c] |
| Fructose-bisphosphate aldolase | fdp[C_c] <=> t3p2[C_c] + t3p1[C_c] |
| Triosephosphate isomerase | t3p2[C_c] <=> t3p1[C_c] |
| Glyceraldehyde 3-phosphate dehydrogenase | pi[C_c] + t3p1[C_c] + nad[C_c] <=> h[C_c] + 13pdg[C_c] + nadh[C_c] |
| Phosphoglycerate kinase | adp[C_c] + 13pdg[C_c] <=> atp[C_c] + 3pg[C_c] |
| Phosphoglycerate mutase | 3pg[C_c] <=> 2pg[C_c] |
| Phosphopyruvate hydratase | 2pg[C_c] <=> h2o[C_c] + pep[C_c] |
| Pyruvate kinase | adp[C_c] + h[C_c] + pep[C_c] => atp[C_c] + pyr[C_c] |
| Pyruvate carboxylase | atp[C_m] + pyr[C_m] + h2o[C_m] + co2[C_m] => adp[C_m] + pi[C_m] + oa[C_m] + 2 h[C_m] |
| Glucose-6-phosphate 1-dehydrogenase | g6p[C_c] + nadp[C_c] => h[C_c] + d6pg [C_c] + nadph[C_c] |
| 6-Phosphogluconolactonase | h2o[C_c] + d6pg[C_c] => h[C_c] + d6pgc[C_c] |
| Phosphogluconate dehydrogenase | nadh[C_c] + d6pgc[C_c] => co2[C_c] + nadph[C_c] + rl5p[C_c] |
| Ribulose-phosphate 3-epimerse | rl5p[C_c] <=> xul5p[C_c] |
| Ribose-5-phosphate isomerase | r5p[c_c] <=> rl5p[c_c] |
| Transketolase | xul5p[c_c] + r5p[c_c] <=> t3p1[c_c] + s7p[c_c] |
| Transketolase | xul5p[C_c] + e4p[C_c] <=> f6p[C_c] + t3p1[C_c] |
| Transaldolase | t3p1[C_c] + s7p[C_c] <=> f6p[C_c] + e4p[C_c] |
| Citrate synthase | oa[C_m] + h2o[C_m] + accoa[C_m] <=> h[C_m] + cit[C_m] + coa[C_m] |
| Aconitate hydratase | cit[C_m] <=> h2o[C_m] + aco[C_m] |
| Aconitate hydratase | h2o[C_m] + aco[C_m] <=> icit[C_m] |
| Isocitrate dehydrogenase (NAD+) | icit[C_m] + nad[C_m] => co2[C_m] + akg[C_m] + nadh[C_m] |
| Isocitrate dehydrogenase (NADP+) | nadp[C_c] + icit[C_c] => co2[C_c] + nadph[C_c] + akg[C_c] |
| α-ketoglutarate dehydrogenase | akg[C_m] + tdpe1[C_m] => co2[C_m] + akge1[C_m] |
| Dihydrolipoamide S-succinyl transferase | akge1[C_m] + 1pse2[C_m] => tdpe1[C_m] +akge2[C_m] |
| Dihydrolipoamide dehydrogenase | coa[C_m] + nad[C_m] +akge2[C_m] <=> nadh[C_m] + 1pse2[C_m] + auccoa[C_m] |
| Succinate-CoA ligase (ADP-forming) | adp[C_m] + pi[C_m] +succoa[C_m] <=> atp[C_m] + coa[C_m] + succ[C_m] |
| Succinate dehydrogenase (ubiquinone) | succ[C_m] + q[C_m] <=> fum[C_m] + qh2[C_m] |
| Succinate dehydrogenase | fadh2[C_m] + fum[C_c] => h[C_m] + fad[C_m] + succ[C_c] |
| Fumarate hydratase | h2o[C_m] + fum[C_m] <=> mal[C_m] |
| Fumarate hydratase | mal[C_c] <=> h2o[C_c] + fum[C_c] |
| Malate dehydrogenase | nad[C_m] + mal[C_m] <=> oa[C_m] + h[C_m] + nadh[C_m] |
| Malate dehydrogenase | nad[C_c] + mal[C_c] <=> h[C_c] +nadh[C_c] + oa[C_c] |
| β-D-Glucose: NADP+1-oxoreductase | bdglc[C_c] + nadp[C_c] <=> h[C_c] + nadph[C_c] + glcn15lac[C_c] |
| Spontaneous reaction or catalyzed by glucose | h2o[C_c] + glcn15lac[C_c] => glcnt[C_c] |
| Gluconokinase | atp[C_c] + glcnt[C_c] => adp[C_c] + h[C_c] + d6pgc[C_c] |
| Phosphoglucomutase | g6p[C_c] <=> glp[C_c] |

Table S3 Metabolite abbreviations and descriptions mentioned in formula.

| Name | Description |
| --- | --- |
| mlt | Maltose |
| glc | α-D-Glucose |
| bdglc | β-D-Glucose |
| atp | ATP |
| adp | ADP |
| g6p | α-D-Glucose 6-phosphate |
| f6p | β-D-Fructose 6-phosphate |
| fdp | β-D-Fructose 1,6-bisphosphate |
| t3p2 | Glycerone phosphate |
| t3p1 | D-Glyceraldehyde 3-phophate |
| pi | Orthophoaphate |
| 13pdg | 1,3-Bisphospho-D-glycerate |
| g1p | α-D-Glucose 1-phophate |
| 3pg | 3-Phospho-D-glycerate |
| 2pg | 2-Phospho-D-glycerate |
| pep | Phosphoenolpyruvate |
| pyr | Pyruvate |
| d6pgl | D-Glucono-1,5-lactone 6-phosphate |
| d6pgc | 6-Phospho-D-gluconate |
| rl5p | D-Ribulose 5-phosphate |
| xul5p | D-Xylulose 5-phosphate |
| r5p | D-Ribose 5-phosphate |
| s7p | Sedoheptulose 7-phosphate |
| e4p | D-Erythrose 4-phosphate |
| [C_c] | Intracytoplasmic |
| [C_m] | Mitochondria |
| oa | Oxaloacetate |
| cit | Citrate |
| icit | Isocitrate |
| akg | 2-Oxoglutarate |
| succoa | Succinyl-CoA |
| succ | Succinate |
| fum | Fumarate |
| mal | (S)-Malate |
| akge1 | α-Ketoglutarate bound to α-ketoglutarate dehydrogenase |
| tdpe1 | (Thiamine diphosphate)-α-ketoglutarate dehydrogenase |
| akge2 | α-Ketoglutarate bound to dihydrolipoyl transsuccinylase |
| lpse2 | (LipS2)-dihydrolipoyl transsuccinylase |
| coa | Coenzyme A |
| accoa | Acetyl-CoA |
| aco | cis-Aconitate |
| glcn15lac | D-Glucono-1,5-lactone |
| glcnt | D-Gluconate |
| qh2 | Ubiquinol |
| q | Ubiquinone |
| nadph | NADPH |
| nadp | NADP^+^ |
| nadh | NADH |
| nad | NAD^+^ |
| fadh2 | FADH2 |
| fad | FAD^+^ |
| h | Protein (energy metabolism) |
| h2o | H_2_O |
| co2 | CO_2_ |

Table S4 Copy number of target genes in overexpression strains identified by quantitative PCR.

| Strains | B36 | OE-*fadE* | OE-*fadA* | OE-*hyp* | OE-*cyp* | OE-*acy* |
| --- | --- | --- | --- | --- | --- | --- |
| Copy muber | 1 | 1.98±0.14 | 1.83±0.11 | 1.89±0.09 | 1.77±0.10 | 2.04±0.19 |

Table S5 Pool sizes of intracellular amino acids for strains OE-*fadE* (overexpressing gene *fadE*), OE-fadA (overexpressing *fadA*) and B36 at steady state. The unit is μmol/gDCW.

| Name | B36 | OE-*fadE* | OE-*fadA* |
| --- | --- | --- | --- |
| Alanine | 28.70±1.04 | 41.91±1.44 | 46.31±1.07 |
| Glycine | 4.82±0.38 | 5.94±0.37 | 6.54±0.15 |
| Valine | 5.07±0.65 | 5.59±0.43 | 5.57±0.76 |
| Leucine | 4.54±0.25 | 6.96±0.29 | 6.98±0.11 |
| Iso-leucine | 0.99±0.04 | 1.10±0.05 | 1.08±0.05 |
| Proline | 1.55±0.04 | 2.33±0.06 | 2.35±0.09 |
| Methionine | 1.47±0.03 | 1.30±0.02 | 1.17±0.03 |
| Serine | 22.38±0.76 | 26.59±0.37 | 25.05±0.90 |
| Threonine | 5.64±0.41 | 8.39±0.55 | 7.33±0.17 |
| Phenylalanine | 0.93±0.01 | 0.94±0.02 | 0.96±0.01 |
| Aspartate | 37.71±1.32 | 45.32±0.65 | 42.70±0.49 |
| Cysteine | 1.02±0.05 | 1.03±0.03 | 1.10±0.03 |
| Glutamate | 92.74±1.93 | 119.48±1.91 | 110.5±2.84 |
| Ornithine | 12.92±0.87 | 14.35±0.64 | 13.77±0.91 |
| Asparagine | 4.38±0.19 | 5.40±0.45 | 4.69±0.21 |
| Lysine | 5.43±0.48 | 7.97±0.52 | 9.26±0.43 |
| Glutamine | 76.84±1.39 | 89.40±1.59 | 78.91±1.70 |
| Histidine | 1.87±0.03 | 1.9±0.03 | 1.97±0.01 |
| Tyrosine | 0.29±0.01 | 0.20±0.01 | 0.21±0.01 |
| total | 309.29 | 386.19 | 366.48 |

Data in the table represent the average values and standard deviations from three replicates.
