# Supplementary material for: Enhancement of fatty acid degradation pathway promoted glucoamylase synthesis in Aspergillus niger
Source: Microb Cell Fact. 2022 Nov 15;21:238. doi: 10.1186/s12934-022-01966-3 (PMC9664828; doi:10.1186/s12934-022-01966-3)
Supplement: Supplementary file 2 — Additional file 2: Supplementary figures. Fig. S1. The relative flux of EMP, PP and TCA pathways in control strain B36 and two recombinant strains at chemostat culture steady state predicted by iHL1210. [file 12934_2022_1966_MOESM2_ESM.docx]

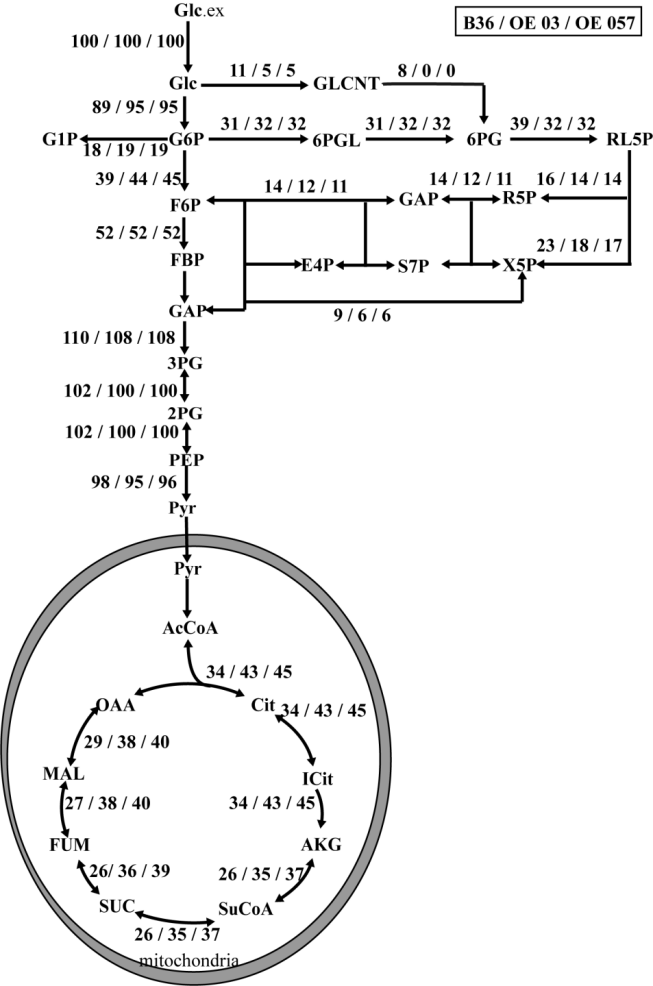


Fig. S1 The relative flux of EMP, PP and TCA pathways in control strain B36 and two recombinant strains at chemostat culture steady state predicted by iHL1210.
